# Interfacial Properties of Miktoarm Star Polymers with a Poly(divinylbenzene) Core

**DOI:** 10.1021/acs.langmuir.5c00288

**Published:** 2025-07-14

**Authors:** Ting-Chih Lin, Mateusz Olszewski, Jiajun Yan, Xiaolei Hu, Krzysztof Matyjaszewski, Philip Taylor

**Affiliations:** a Department of Chemistry, 6612 Carnegie Mellon University, Pittsburgh, Pennsylvania 15213, United States; b School of Physical Science and Technology, 387433 ShanghaiTech University, Pudong, Shanghai 201210, China; c Department of Molecular Physics, Faculty of Chemistry, Lodz University of Technology, Żeromskiego 116, Łódź 90-924, Poland; d Syngenta, Jealott’s Hill International Research Centre, Bracknell, Berkshire RG42 6EY, U.K.

## Abstract

The interfacial properties of miktoarm star polymers composed of poly­(divinylbenzene) (PDVB) cores with poly­(ethylene oxide) (PEO) hydrophilic arms and poly­(*n*-butyl acrylate) (PBA) or poly­(lauryl acrylate) (PLA) hydrophobic arms at the oil/water interface are reported. The kinetics of miktoarm star polymer adsorption from the oil phase depended on the polymer concentration. This suggested that the rate-determining step was the adsorption and penetration of the polymer onto and through the interface. This was attributed to the desolvation of the polymer arms from the oil phase being less facile than that of similar star polymers with PEO arms alone from the aqueous phase. PEO star polymers showed diffusion-controlled kinetics, and as such, the adsorption and so forth were facile and rapid compared to the rate of diffusion to the interface. The interfacial oscillatory dilatational rheology of the polymers adsorbed at the interface was dependent on the relative molecular weights or lengths of the arms. In the case where both arms showed similar contour lengths, the interfacial rheological response was strongly frequency dependent, suggesting that the polymer adsorption/desorption was relatively facile on the time scale of the oscillation. Polymers in which the hydrophilic arms were longer than the hydrophobic arms showed a relatively frequency-independent response. This was attributed to the hydrophobic arms effectively increasing the size of the hydrophobic core in conjunction with the penetration of the hydrophilic arms through the interface into the aqueous phase, thus pinning the polymer at the interface.

## Graphical abstract

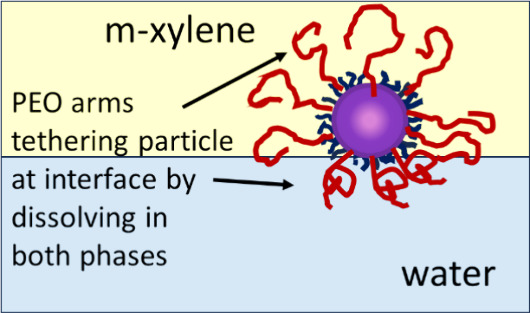

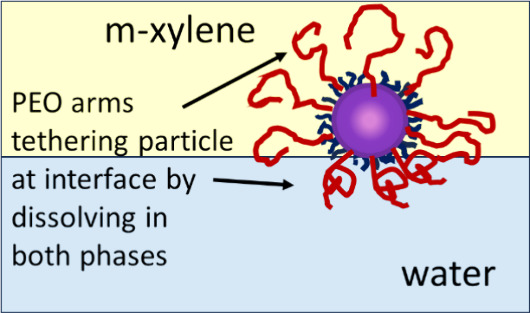

## Introduction

The adsorption of nanoparticles, such as star polymers, has gained interest due to their tunable properties.^1–8^ We previously reported the oscillatory and relaxation investigation of interfacial rheology of star polymers with low grafting density of poly­(ethylene oxide) arms and hydrophobic poly­(divinylbenzene) cores.^9^ In this report, we expand on the structure of star copolymers by incorporating mixed arms: hydrophilic poly­(ethylene oxide) (PEO) arms and hydrophobic poly­(*n*-butyl acrylate) (PBA) or poly­(lauryl acrylate) (PLA) arms. These stars were prepared by Atom Transfer Radical Polymerization (ATRP).^10–14^ There are two routes for the synthesis of stars: core-first and arm-first approaches. The former method employs a multifunctional core with embedded ATRP initiators such as α-bromoisobutyrate esters, followed by growth of the arms controlled by ATRP copper complexes.^15–21^ They generally produce arms with identical arm chemical structures, though one can stop the growth of some arms and prepare stars with bimodal molecular weight distributions. The arm-first approach employs macroinitiators or macromonomers that are cross-linked in the presence of a divinyl cross-linker, such as divinylbenzene, using ATRP copper catalysts and, optionally, additional initiators when employing macromonomers.^22–26^ Macroinitiators can be prepared by ATRP of various vinyl monomers, such as (meth)­acrylates, (meth)­acrylamides or styrene, as relatively short polymer chains with ATRP dormant chain ends that can be subsequently activated by ATRP catalysts in the cross-linking step with divinyl compounds to generate stars.^27–30^ Alternatively, polymers prepared by other mechanisms, such as anionic ring-opening polymerization, can be esterified to form α-bromoisobutyrate to be active in ATRP.^31^ Using chemically different macroinitiators or macromonomers opens the pathway to miktoarm star polymers. In general, the successful synthesis of star polymers by the arm-first approach requires an appropriate molar ratio of divinyl cross-linker to macroinitiators (ca. 10:1).^25^ Excess cross-linkers lead to coupling of star cores and even macroscopic gelation, whereas an insufficient amount of cross-linkers will form just linked chains rather than hairy nanoparticles.^18^ It was confirmed by 2D-chromatography (HPLC-SEC) that mixing two (or more) different macroinitiators resulted in a miktoarm star (with mixed arm composition) rather than a mixture of homoarm stars with two different compositions.^25,32,33^ In this work, we focused on the synthesis of amphiphilic miktoarm stars with hydrophilic PEO arms and hydrophobic PBA or PLA arms

Star polymers are known to adsorb at oil/water interfaces and have been used as emulsion stabilizers.^1,3,9^ The adsorption of polymers at interfaces has been studied using various techniques, including interfacial tension and interfacial dilatational rheology.^34–40^ Their adsorption leads to a decrease in the interfacial tension, dependent on their structure and composition. For example, the adsorption of homopolymer stars with a PDVB core and PEO arms reduced the interfacial tension at both the m-xylene/water and n-dodecane/water interfaces, where the extent and rate of the reduction were dependent on the molecular weight of the PEO arms.^9^ Miktoarm stars appear to show greater reduction in the interfacial tension, in some cases to less than 4.4 mN m^–1^ at the m-xylene/water interface compared to 10–15 mN m^–1^ in the case of homopolymer PEO stars.^3^ Interfacial tension measurements are useful in the study of the adsorption of star polymers, but further information may be gained using interfacial dilatational rheology. In these measurements, the variation in interfacial tension with a change in an interfacial area allows the interfacial dilatational moduli to be determined.^35–37^ These have been used in a number of studies to investigate the adsorption of star polymers and similar nanoparticles at the o/w interface, including homopolymer stars with PDVB cores and PEO arms.^1,3,9,40^ The magnitude and frequency dependence of these moduli, in conjunction with the adsorption kinetics of the polymers, give insights into the strength and reversibility of the adsorption at the interface. For example, homopolymer stars adsorbing from the aqueous phase show diffusion-controlled kinetics and some degree of reversibility when adsorbed at the o/w interface.^9^ The kinetics and reversibility of miktoarm polymers is not yet well understood and will be considered here. In this study, we have combined interfacial tension and interfacial dilatational moduli measurements to investigate the effect of varying the molecular weights of PBA and PEO arms on PDVB cores for miktoarm star polymers each containing equal masses of the two arm chemistries.

## Experimental Section

### Polymer Preparation

#### Materials

Divinylbenzene (DVB, 80%, Aldrich), *n*-butyl acrylate (BA, 99%, Acros), and lauryl acrylate (LA, 98%, TCI) were purified by passing through a column of basic alumina. Poly­(ethylene glycol) methyl ether (*M*_n_ = 5000, Aldrich) was used to prepare poly­(ethylene oxide)-based macroinitiators by base-catalyzed transesterification using a previously published procedure.^9,25,41^ Copper­(II) bromide (CuBr_2_, 99%), tin­(II) 2-ethylhexanoate (Sn­(EH)_2_, 95%), *N,N*-dimethylformamide (DMF, 99%), tris­(2-pyridylmethyl)­amine (TPMA, 99%, KOEI), tris­(2-dimethylaminoethyl)­amine (Me6TREN, 99%, KOEI), anisole (99%, TCI), ethyl 2-bromoisobutyrate (EBiB, 98%, Aldrich), and copper wire were used as received. M-xylene (anhydrous 99% Aldrich) and n-dodecane (anhydrous 99% Aldrich) were stored over fumed silica (Aerosil OX-50) to remove surface active impurities.

#### Nuclear Magnetic Resonance Spectroscopy (NMR)

^1^H NMR spectra were acquired with a Bruker Avance 500 MHz using CDCl_3_ as the solvent.

#### Gel Permeation Chromatography (GPC)

Molecular weights of macroinitiators and stars were determined by GPC The GPC system was equipped with PSS columns (SDV 10^2^, 10^3^, and 10^5^ Å), Waters refractive index detector, and PSS SLD9000 light scattering detector with THF as eluent at a flow rate of 1 mL/min at 35 °C. Toluene was used as an internal standard and calibrated vs linear PMMA standards.

#### Poly­(*n*-butyl acrylate) Macroinitiator Synthesis

Into a Schlenk flask, BA (18 mL, 65 equiv), EBiB (0.3 mL, 1 equiv), CuBr_2_ (60 mg, 0.013 equiv), Me_6_TREN (43 μL, 0.1 equiv), copper wire (1 mm × 5 mm), and anisole (38 mL) were added. The solution was deoxygenated, then allowed to react at room temperature until desired monomer conversion was achieved. Monomer conversion was monitored by ^1^H NMR.

#### Poly­(lauryl acrylate) Macroinitiator Synthesis

Into a 25 mL Schlenk flask, LA (4.5 mL, 40 equiv), EBiB (72 μL, 1 equiv), CuBr_2_ (5 mg/mL stock solution in DMF, 3.7 mg, 0.04 equiv), Me_6_TREN (20 mg/mL stock solution in DMF, 15.3 mg, 0.16 equiv), and 4 mL anisole were added. The solution was sparged with nitrogen for 15 min, then lowered into a 15 × 8 cm UV light reaction chamber (λ=365 nm, 4.2 mW). The flask was positioned 4 cm away from the light source. Monomer conversion was monitored by ^1^H NMR, and the reaction was quenched by opening to air once the desired conversion was reached.

#### Polymer Star Synthesis

In general, a 10 mL Schlenk flask was charged with divinylbenzene (DVB, 15 equiv) and desired macroinitiator arms (total 1 equiv), tris­(2-pyridylmethyl)­amine (TPMA, 0.1 equiv) and copper­(II) bromide (CuBr_2_, 0.01 equiv) stock solutions in DMF, and anisole. The solution was purged with nitrogen for 20 min, then a deoxygenated solution of tin­(II) ethyl hexanoate (Sn­(EH)_2_, 0.2 equiv) in anisole was added to the solution under nitrogen protection. The flask was sealed and submerged in a 90 °C oil bath. DVB conversion was tracked by NMR, and the reaction was quenched by opening to air upon reaching the desired DVB conversion.

#### Interfacial Tension

Measurements of the interfacial tension were made on a DataPhysics ODG20 oscillating drop tensiometer at 20 ± 1 °C. Water droplets of ca. 20 μL volume were formed on the end of square-ended 0.50 or 1.65 mm outer diameter syringe needles within the solution of the star polymer in the oil phase. The oil phase was contained within a 2 × 2 × 2 cm glass cuvette that had been previously cleaned with chromic acid. The droplet was imaged using the ODG20 camera, and the interfacial tension was determined through profilometry using the instrument tracking function. The droplet tracking was set running, and the droplet was then formed as quickly as possible. The measurement was continued until the interfacial tension had reached a steady value. m-Xylene gave an interfacial tension against water of 37 mN m^–1^ and n-dodecane gave 52 mN m^–1^.

#### Interfacial Dilatational Measurements

The dilatation measurement was carried out on the equilibrated droplets formed in the interfacial tension measurements. The droplet was subjected to a sinusoidal variation in volume using the piezo-electric actuator system of the ODG 20. The actuator was set to an amplitude in the range 0.05–0.1 mm to give a fractional droplet area change of around 2.5–5%. The oscillations were made at seven frequencies between 0.01 and 1 Hz, and five complete cycles were performed at each frequency. The droplet profile was recorded using the instrument camera, and 200 images were recorded at each frequency. The images were analyzed to give the variation in interfacial tension (γ) and droplet area (A) as a function of time, and both were found to be sinusoidal. The two responses were then subjected to a Fourier transform analysis to give the interfacial complex, storage, and loss moduli (ε*, ε’ and ε” respectively).

The complex modulus is given by the change in interfacial tension with the relative change in the interfacial area (A/Ao, where Ao is the average interfacial area), described by eq 1.

$$\varepsilon^{*}=\frac{d\gamma }{d\ln\left(\frac{A}{A_{o}}\right)}=A_{o}\frac{d\gamma }{dA}$$ 1

The interfacial area (A­(t)) of the droplets varied sinusoidally with an amplitude of A about its mean value, A_o_, with time (eq 2)­

$$A\left(t\right)=A_{o}+\mathrm{\Delta Acos}\left(2\pi ft\right)$$ 2

Each image was analyzed using the instrument software to give the variation in interfacial tension with (γ (t)). The amplitude of the interfacial oscillation was determined (Δγ) and the variation of the interfacial tension during the oscillation is given by eq 3 ^42^

$$\gamma \left(t\right)=\gamma_{o}+\Delta \gamma \cos\left(2\pi ft+\phi \left(f\right)\right)$$ 3

Where ϕ­(f) is the phase angle in radians between of the sinusoidal variations in area and interfacial tension. The instrument software applies a Fourier transform to the variation in interfacial tension and area to extract ε’(f) and ε”(f) at each individual frequency (eqs 4 and 5).

$$\varepsilon^{\prime }\left(f\right)=\Delta \gamma \left(f\right)\frac{A_{o}}{\Delta A}\cos\phi \left(f\right)$$ 4

$$\varepsilon^{\prime \prime }\left(f\right)=\Delta \gamma \left(f\right)\frac{A_{o}}{\Delta A}\sin\phi \left(f\right)$$ 5

Repeat measurements on selected samples suggested an error of ± 10% between repeated samples.

#### Emulsion Stability

10% v/v oil-in-water emulsions were prepared by premixing 1 mL of oil phase with 9 mL of 0.1% polymer solutions and shaken by hand to give coarse droplets. These were further emulsified by subjecting the system to ultrasonic agitation for 30 s using an ultrasonic probe with a 3 mm tip. The samples were stored at 20 °C and periodically sized using a Malvern 2000 laser diffraction system, making three measurements at each time point.

#### Dynamic Light Scattering

The hydrodynamic sizes of the polymers were determined using dynamic light scattering using either a Malvern Nano S (Stars 52, 55, and 210) and/or a Malvern Zetasizer (PLA–PEO analog). The measurements were made at 20 °C in either m-xylene or o-xylene. Due to a small population of larger scattering units (probably resulting from some degree of polydispersity and possible aggregation and/or coupling of particles), the intensity-derived size distributions were highly skewed to large sizes. To circumvent this, the data was transformed into volume distributions using the Malvern Nano S or Malvern Zetasizer software.

## Results and Discussion

Three star polymers based on PEO and PBA (PBA–PEO) are shown schematically in Figure 1. The relative numbers of arms are correct and are of the correct relative lengths based on the contour lengths of the polymers based on data reported by Wang et al., who gave the following relationships for PEO and PAA (poly acrylic acid) contour lengths (L_c,PEO_ and L_c,PAA_ respectively) for chains of the degree of polymerization n (eq 6):^43^

$$L_{c,PEO}=0.3683n,L_{c,PAA}=0.2475n$$ 6

**Figure 1:**
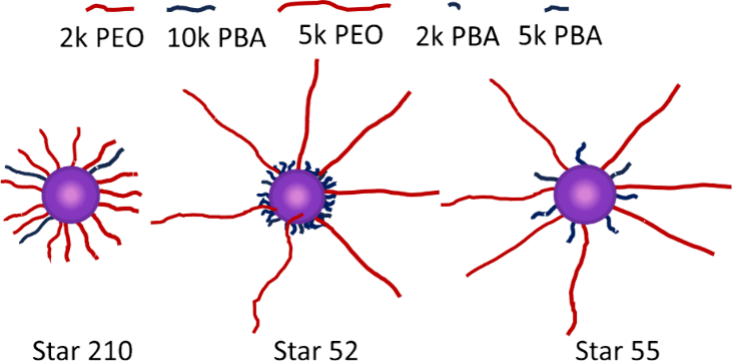
Schematic representation of the three PBA–PEO analogs showing the relative extended lengths of the two arm chemistries.

Star 210 polymer was comprised of arms of similar length (ignoring any conformational differences for now), the contour length of the 2k PEO being 16.8 nm and that of the 10k PBA backbone being 19.2 nm. It was also the most hydrophilic overall in terms of relative numbers of arms, having five times as many PEO arms as PBA arms, prepared with equal masses of the two polymer arms. Star 52 contained two and a half times as many PBA (3.8 nm) arms as PEO, but these were of the order of one-11th the length of the PEO arms. Star 52 was comprised of the relatively fewest number of hydrophilic arms and had the most pronounced hydrophobic core, the short, higher density layer of PBA becoming essentially part of the core, and was likely to be the most hydrophobic of the three. Star 55 contained equal numbers of 5k PEO (contour length 42 nm) and 5k PBA (contour length 9.6 nm) arms, with the PEO arms being approximately 4.4 times the length of the PBA. Star 55, with its equal number of hydrophilic and hydrophobic arms, was of intermediate hydrophobicity overall. It could be argued that the relative hydrophobicities could be assessed based on the number of EO and BA monomer units. However, since all three systems were prepared using equal masses of the two monomers, they all have the same EO:BA molar monomer ratio of 2.9:1, and it might be assumed that all three stars have the same overall relative hydrophobicity. It will be shown that these star polymers show very different behavior at the O/W interface. This indicates that the spatial distribution of the monomeric units within the star polymer as a whole and the lengths of the arms have a significant effect on the overall properties of the stars.

In addition to the differences in length, the relative graft densities of the arms on the three polymers would be expected to affect the nature of their adsorption at the O/W interface. The PDVB core made up 25% of the total molecular weight in all four polymers. The core radii were estimated from the mass average molecular weight assuming a density of 1.02 g·cm^–3^ for PDVB. The number and area of arms were also calculated from the mass average molecular weight and are listed in Table 1. Stars 52 and 210 showed similar graft densities of 2.3- 2.8 nm^2^ per arm while that of Star 55 was 4 nm^2^. The lowest graft density was seen with the PLA–PEO analog at 6.6 nm^2^ per arm, this was likely due to the increased length of the lauryl side chains on the PLA arm causing poorer packing.

**Table 1:** Number of Arms and Area per Arm for the PBA–PEO and PLA–PEO Star Polymers Based on Weight Average Molecular Mass (*M*_w_) Obtained by GPC^a^

polymer	*M*_w_/g mol^–1^	M_wcore_/g mol^–1^	r_core_/nm	nPEO	nPBA or nPLA	A_arm_/nm^2^
Star 55	88,900	22,200	2.1	6.7	6.7	4.0
Star 52	82,800	20,700	2.0	6.2	15.5	2.3
Star 210	74,100	18,500	1.9	13.9	2.8	2.8
PLA–PEO	25,000	6200	1.3	1.9	1.6	6.6

a The area per arm (A_arm_) is based on the total number of arms per particle. Confer SI for the more detailed discussion on absolute molecular weights, number of arms and arm densities.

The sizes of the polymers were determined by dynamic light scattering, and the derived volume size distributions are shown in Figure 2. All three PBA–PEO polymers showed a major peak corresponding to the primary particle size, while Stars 52 and 210 showed a small peak at higher diameters of around 50–200 nm, possibly due to some aggregation or interparticle coupling resulting from their chain end functionality. Star 55 and Star 52 showed volume average diameters for this peak of 21.9 and 23.2 nm, respectively. The volume average diameter of Star 210 for the primary peak was somewhat lower at 13.8 nm. The diameter of the particle was strongly dependent on the molecular weight of the PEO chain, with the particles containing the 5k PEO showing similar sizes that were significantly larger than Star 210 with the 2k PEO. A comparison of the particle size with the 5k PEO contour length shows that in m-xylene, the polymer is in some form of a random walk conformation rather than a fully extended conformation, and so maybe better described by the radius of gyration (*R*_g_). The chain is not expected to be fully collapsed due to poor solvency since the Flory–Huggins parameter of PEO in the very similar solvent of o-xylene was 0.38–0.44 (albeit at 70 °C).^44^

**Figure 2:**
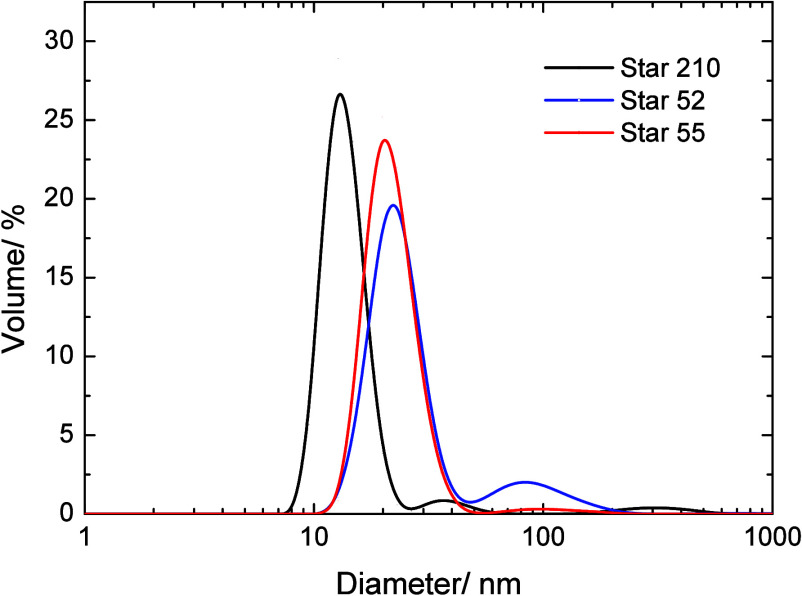
Dynamic light scattering size distributions of the three PBA/PEO analogs transformed to volume percent. Measurements made in m-xylene at 20 °C.

The radius of gyration of PEO depends on the solvency of the solvent and has been modeled by Hezaveh et al., and analysis of their data for low molecular weights up to 1892 shows a power law dependence on the molecular weight (*M*_w_) of the polymer and the solvency.^45^ No aromatic solvents similar to m-xylene were included in Hezaveh’s report and in order to get an indication of the magnitude of the radius of gyration it was calculated using eq 7 for PEO in water and in chloroform (described elsewhere as a good solvent for PEO^54^)^45^

$$R_{g}=cM_{W}^{n}$$ 7

Where c = 0.015 and n = 0.62 for water and c = 0.018 and n = 0.55 for chloroform. On this basis, the radii of gyration of 2k and 5k PEO were of the order of 2 and 3 nm, respectively in water and 1 and 2 nm in chloroform, respectively. The radii of the PDVB core of the PBA–PEO miktoarm star polymers were 2 nm such that the 5k PEO hydrodynamic thickness in Stars 52 and 55 were 10 and 9 nm, respectively, while for Star 210 with a core radius of 1.9 nm the 2k PEO thickness was of the order of 5.5 nm. While the radius of gyration calculated for water or chloroform as the solvent was simply to indicate a typical size in a reasonable solvent, it is clearly significantly smaller than the layer thicknesses obtained from dynamic light scattering. This suggests that the PEO on the star polymers is stretched out from its natural conformation in a good solvent, possibly either due to a conformational restriction imposed by the tethering of one end of the chain to the core or to steric hindrance from the branched PBA chains. The PLA–PEO analog in o-xylene showed a volume mean diameter of 15.4 ± 1.1 nm and a core radius of 1.3 nm, giving a comparable layer thickness of 6.4 nm. The thickness of the PEO layer on the 5k PEO homopolymer (mean diameter 15 nm and core radius 2.3 nm) was previously reported to be 5.2 nm. Both are lower than that found for the two PBA-5k PEO analogs. The lack of any hydrophobic arms in the homopolymer and the low number of PLA in the PLA–PEO appear to be causing a less extended conformation.

All three PBA–PEO star polymers dissolved to form clear solutions in m-xylene. Some spontaneous emulsification was seen with 0.2% Star 52 at the m-xylene/water interface when left for two hours, Figure 3. The suspended water droplet became surrounded by a hazy region of droplets, and to minimize this effect on the measurements, the length of time for equilibrating the droplets was reduced, and the interfacial data for Star 52 should be recognized as being close to equilibrium rather than completely at equilibrium. This will have had some effect on the data but is not expected to have had a significant effect on the results and their interpretation. This spontaneous emulsification has been described by Huang et al., who attributed it to the budding of oil droplets at the interface arising from the adsorption of the polymer, allowing a localized increase in interfacial area.^1^ These would then detach into the aqueous phase to form an oil-in-water emulsion. Second, the adsorptions of Stars 52 and 55 and of the PLA–PEG analog at the interface were sufficiently strong such that when an equilibrated droplet used in the interfacial measurements was shrunk by sucking out the water, the droplet became wrinkled as the particles did not readily desorb and the interface wrinkled to maintain a large interfacial area despite the drop in volume, Figure 3. It was uncertain whether the Star 210 polymer showed this effect since it was found to be difficult to shrink the droplet without it detaching from the needle owing to its low interfacial tension. However, no wrinkling was seen in the limited shrinkage of the droplets before detachment.

**Figure 3:**
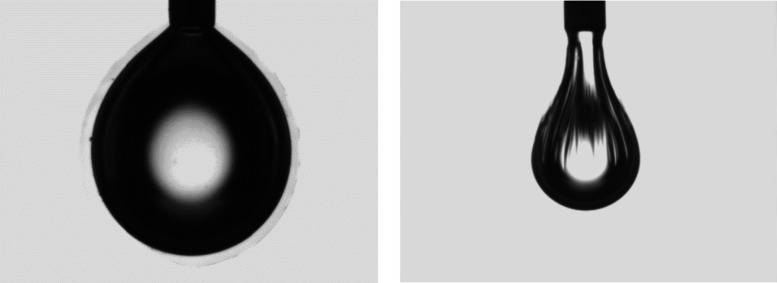
Left- 0.2% Star 52 in m-xylene showing haze formation around the droplet. Right- Deflated drop of 0.025% Star 55 in m-xylene after equilibration and rheology measurement.

The error in pendant drop profilometry depends on the extent of the droplet deformation from spherical, and the deformation seen here varied between the polymers. A full description of the droplet shapes and the accompanying accuracy of the measurements is given in the Supporting Information. That analysis indicated the error to be within an error bound of ± 0.5 mN m^–1^. The solutions of the three PBA-based polymers in m-xylene showed significant differences in interfacial tension against water, the lowest being for Star 210 at ca. 4 mN m^–1^, Star 52 gave an intermediate value of ca. 10 mN m^–1^ and Star 55 the highest at ca. 16 mN m^–1^, Figure 4. Both Star 210 and 55 showed little dependence of the interfacial tension with concentration, whereas Star 52 showed a small initial decrease at low concentration. However, this may be due to very slow approaches to equilibrium seen with this polymer.

**Figure 4:**
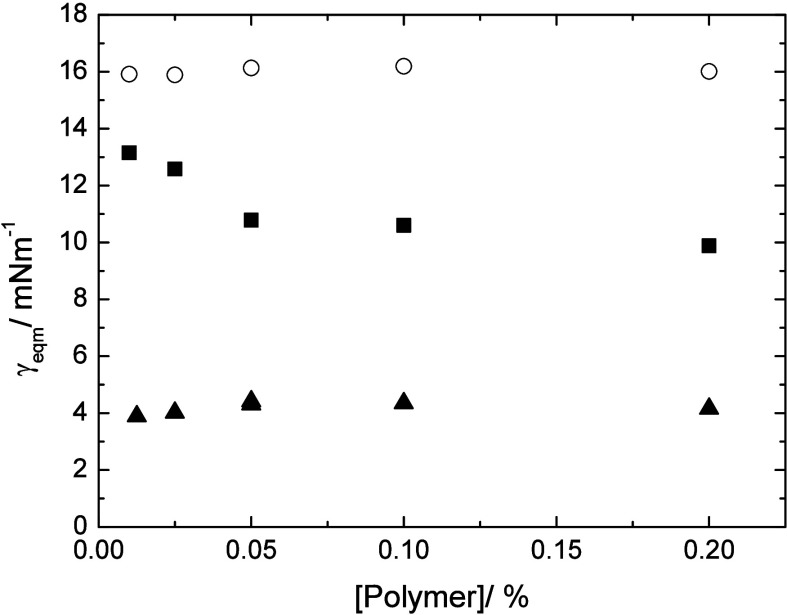
Concentration dependence of the interfacial tension of the star polymers at the m-xylene/water interface (error ± 0.5 mN m^–1^), Star 55-circles, Star 52-squares, Star 210-triangles.

Differences in the time dependence of the interfacial tension were also observed, Stars 55 and 52 showed a fast initial reduction in interfacial tension followed by a slow final approach (Figure S4, Supporting Information). Star 210 showed a slower initial decrease than these two polymers but appeared to reach an equilibrium value more rapidly and did not show a slow approach to equilibrium (Figure 5). The PLA–PEO system in n-dodecane (Figure S3, Supporting Information) showed a similar rapid initial decrease to Star 55 but followed by a less pronounced slow approach to a constant value, the equilibrium interfacial tension varied little with concentration (Figure S3, Supporting Information).

**Figure 5:**
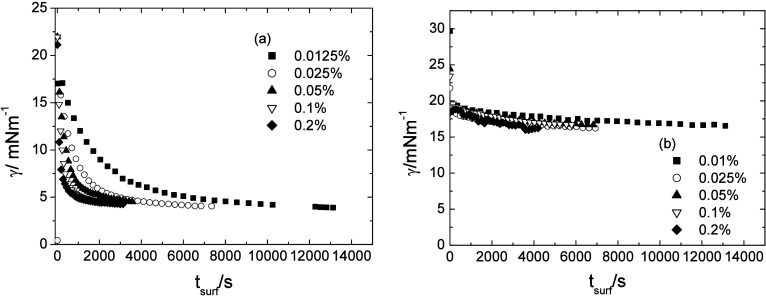
Time dependence of the interfacial tension for miktoarm star polymers at the m-xylene/water interface, left- Star 210 (a), right Star 55 (b). Error in interfacial tension ± 0.5 mN m^–1^.

The differences in the equilibrium tension between the different polymers complicate simple comparison between the time-dependent interfacial tensions, and the data is more readily visualized as plots of the reduced interfacial tension (γ_red_) versus surface age (t_surf_), where γ_red_ is given by eq 8.^39^

$$\gamma_{red}=\frac{\gamma_{t}-\gamma_{e}}{\gamma_{o}-\gamma_{e}}$$ 8

Where γ_t_, γ_o_ and γ_e_ are the interfacial tensions at time t, of the clean interface (37 mN m^–1^) and the equilibrium or final value. Plots of γ_red_ vs t_surf_ are shown in Figure 6 for 0.1% solutions of Stars 52, 55, and 210 in m-xylene, alongside that for 0.1% of a 5k PDVB–PEO homopolymer star in water adsorbing at the m-xylene/water interface. Star 55 showed a rapid initial decrease in interfacial tension but then showed a much slower final approach to equilibrium. Star 52 also showed an initial rapid fall in interfacial tension, but the slower approach to equilibrium began at a higher reduced interfacial tension of around 0.4 compared to 0.2 for Star 55 and showed overall slower kinetics than Star 55. Star 210 showed somewhat different behavior in that although it also showed a rapid initial decrease with a slowing down occurring around a γ_red_ of 0.4, it still reached equilibrium (γ_red_ = 0) within 2000 s compared to 5000–6000 s required for Stars 55 and 52. The 5k PEO homopolymer showed a similar hydrodynamic size (volume mean diameter of 14.4 nm) to Star 210 (15.4 nm) and both polymers showed similarly fast reduction in interfacial tension. Comparison Stars 52 and 55 with the 5k PEO homo polymer (with a comparable molecular mass of 1.2 × 10^5^g mol^–1^) highlights a significant difference in rates between the mikto and homo variants.

**Figure 6:**
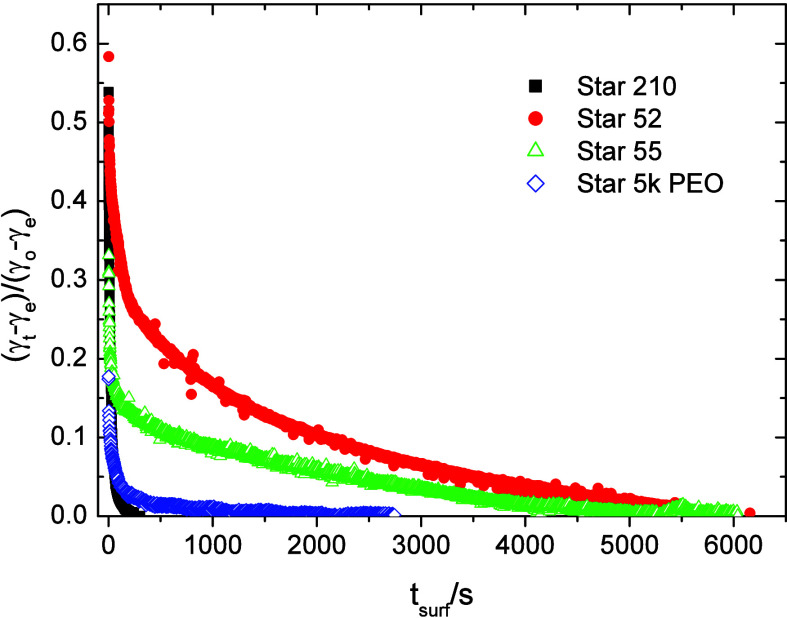
Plots of the reduced m-xylene/water interfacial tension versus time for 0.1% solutions of Stars 52, 55, and 210 in m-xylene and 5k PEO homopolymer star in water. Error in interfacial tension ± 0.5 mN m^–1^.

The approach to equilibrium of the interfacial tension for all four miktoarm polymers could be normalized by multiplication of the surface age by the polymer concentration, leading to an acceptable collapse of the interfacial tension at different concentrations onto a single master curve. However, it was reported in a previous publication that the adsorption of homopolymer PEO stars with PDVB cores was diffusion-controlled, which required a different normalization of the data.^9^ Under diffusion-controlled adsorption, the characteristic time scale of the adsorption process is described by a normalization of the surface age by the square of the concentration.^9,46^ Applying this normalization method to the data for Stars 52, 55, and 210 did not lead to a collapse of the data onto a single curve. A comparison of the two modifications is shown in Figure 7 for Star 210, which most clearly shows the difference between the two approaches. Similar collapse using the simple multiplication by the concentration (Figure S5 Supporting Information) was found for both the adsorption of Stars 52 and 55 (the latter is not shown) and the star polymer with PEO and PLA arms at the water/n-dodecane. This showed that the effect was not specific to the adsorption from aromatic m-xylene but was also seen when the adsorption was from an aliphatic oil phase. Similarly, it was not specific to the poly­(n-butyl acrylate) chemistry but also included poly­(lauryl acrylate).

**Figure 7:**
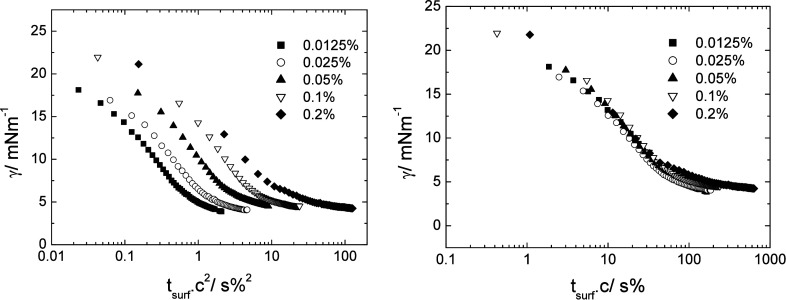
Normalization of the interfacial tension time versus time for Star 210 by the square of the concentration (c^2^) (left) and by the concentration (c) (right) at the m-xylene/water interface. Error in interfacial tension ± 0.5 mN m^–1^.

For comparison, the behavior of the homopolymer 5k PEO star adsorbing from the aqueous phase at the water/m-xylene interface obtained previously is shown in Figure 8. A good collapse of the data was seen when normalized by the square of the concentration, but a much poorer collapse when normalized by concentration alone.

**Figure 8:**
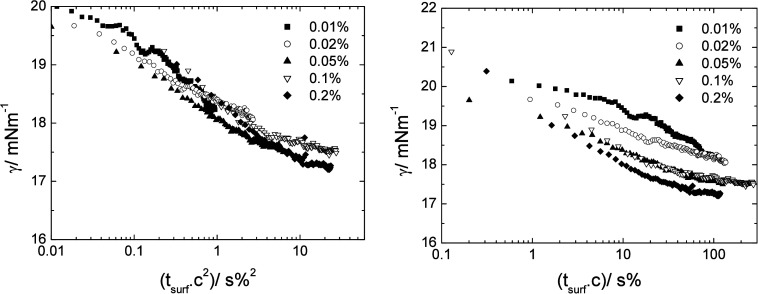
Interfacial vs surface age for 5k PEO homopolymer star adsorbing at the m-xylene/water interface from the aqueous phase. Left- surface age modified by the square of the concentration for diffusion control (t_surf_c^2^). Right- surface age modified by the concentration for adsorption rate control (t_surf_c). Error in interfacial tension ± 0.5 mN m^–1^.

Diffusion-controlled adsorption kinetics have been modeled by determining the thickness of a layer of solution at the interface that contains just sufficient adsorbate to saturate the interface at that concentration.^46^ The time scale of the adsorption is determined by the time required for the adsorbing outermost molecules in this layer to diffuse to the surface or interface, and the interface reaches saturation. This essentially assumes that the actual adsorption process onto the interface is fast compared to the diffusion time, such that the diffusion process is the rate-determining process. However, this is clearly not the case with the miktoarm star polymers used here.

The normalization of the data by the concentration alone suggests that the adsorption process itself is sufficiently slow to be the limiting step. Under these conditions, the concentration of the adsorbing particle remains essentially constant near the interface as the rate of diffusion is sufficiently high to replenish any loss due to the much slower adsorption process. Thus, the rate of adsorption will now be given by the rate at which a particle adsorbs onto the interface, which will be proportional to the concentration of particles. The behavior of the Star 210 may be rationalized in these terms. The slow adsorption process may result from either an activation energy for the adsorption of the particle or a facile desorption of the particle, leading to a reduced net rate of adsorption. Lin et al. proposed Equation 9 for the rate of change in surface excess with concentration for the adsorption of long-chain alcohols with both adsorption and desorption of molecules.^47^

$$\frac{d\Gamma }{dt}=\left[\beta \exp\left(-\frac{E_{a}}{RT}\right)\right]c_{s}\left(\Gamma_{\infty }-\Gamma \right)-\left[\alpha \exp\left(-\frac{E_{d}}{RT}\right)\right]\Gamma$$ 9

*E*_a_ and E_d_ are activation energies to adsorption and desorption, Γ is the surface excess at a surface concentration c_s_ and Γ_∞_ is the surface excess at saturation. α and β are constants. At a given interfacial tension (and, hence, equal surface excess) the characteristic time scale of the rate of change in surface excess is not simply proportional to the concentration, the desorption term being independent of concentration. Taking (dΓ/dt)^−1^ to be representative of the time scale of the overall process, the time scale does not scale with 1/c owing to the concentration-independent term. On this basis, it is taken that the star polymer adsorption kinetics is not a result of any facile desorption modifying the process.

Liggieri et al. proposed that rather than adsorbing following purely diffusion-controlled kinetics, surfactants adsorb via a mixed diffusion-controlled/adsorption activation energy kinetics.^48^ In this approach, the kinetics of the adsorption are represented by an activation energy, *E*_a_, which acts to modify the diffusion coefficient, D, of the surfactant to give an effective diffusion coefficient, D_eff_ given by eq 10.

$$D_{eff}=D\exp\left(\frac{-E_{a}}{kT}\right)$$ 10

The mixed model shows that surfactant adsorption follows diffusion-controlled kinetics, but at a rate modified by the activation energy to adsorption and as such the time scale would be normalized by the square of the concentration. The homopolymer PEO star appears to follow this mechanism since the square of the concentration normalizes the time scale, but the rate also depends to some extent on the nature of the oil phase and the composition of the polymer.^9^ For instance, the absolute kinetic rate for a given polymer was slower with an oil phase of m-xylene than with n-dodecane. For the kinetics to show diffusion-controlled time scale normalization, the rates of the two processes must be sufficiently close such that one or the other of the two processes does not completely dominate the behavior.

In the case of the miktoarm polymers, the activation energy against adsorption must be so high that it completely dominates the adsorption time scale. The activation energy (*E*_a_) dependent rate would be given by eq 11:

$$Rate\propto c\exp\left(\frac{-E_{a}}{RT}\right)$$ 11

The pre-exponential factor includes the concentration and leads to the time scale scaling with concentration. The activation energy may depend on the fractional surface coverage or interfacial tension but would be independent of concentration. Since the activation energy for any given interfacial tension is constant, the data would scale with concentration.

The time dependence of star polymers appears to depend on the phase from which it is adsorbing. This difference arises from differences in rate of adsorption and associated activation energy and is not due to differences in the viscosities of the respective phases. The diffusion coefficient of the star polymers in either liquid will depend on the viscosity. However, the viscosities of water and m-xylene are similar; at 20 °C, the viscosity of water is 8.9 × 10^–4^ Pa s compared to 6.2 × 10^–4^ Pa s for m-xylene. Although the m-xylene is less viscous, which would promote faster diffusion, the difference is too small to account for the change in kinetics seen here. The cause of the difference is then likely to be a result of the relative rates of the desolvation and resolvation of the polymer arms in the different phases required for the PEO chains to adsorb and penetrate from their solution into the second phase. PEO is well solvated by m-xylene, and the interfacial tension kinetics previously seen for the homopolymer PEO star adsorbing onto and penetrating the m-xylene could be explained in terms of the transfer of the PEO from the water to the m-xylene.^9^ In this case, this process was sufficiently fast for the process to be primarily controlled by diffusion to the surface. For the miktoarm adsorption from the m-xylene, the rate of desolvation of the PEO arms and their subsequent penetration and rehydration into the aqueous phase would appear to be much slower, leading to the observed kinetics.

The homopolymer PEO stars previously reported^9^ had low grafting densities of 2k and 5k PEO arms (16 or 25), which may be thought to facilitate rapid dehydration and solvation by the oil phase. In these polymers, the area per grafted chain was of the order of 3.6–4.1 nm^2^, similar to that of Star 55 (4 nm^2^). Despite similar graft densities, quite different rates of approach to equilibrium were seen for these three polymers. The higher graft density on Star 52 was 2.8 nm^2^ per arm and this greater density may have contributed to the very slow kinetics through a reduction of the rate of the exchange of m-xylene for water within the corona of grafted arms. These results suggest that while graft density may affect the kinetics of adsorption, it is not the only factor.

An alternative explanation may arise from the hydrophobic PBA or PLA arms requiring conformational changes at the interface, to accommodate the miktoarm star at the interface in its lowest interfacial energy state. The process would be more convoluted in the case of PLA arms compared to that of PBA arms due to the longer pendant chains showing greater steric hindrance that would affect any required conformational changes, although that effect would be reduced by the low graft density on the PLA–PEO polymer (6.6 nm^2^). The differences in kinetic time scale scaling have not previously been reported and require further work to confirm whether the suggested hypotheses are correct, but they are generally consistent with the data obtained here.

The interfacial moduli were found to be independent of the fractional area changes applied in the measurements (<0.05). All four polymers showed a sinusoidal interfacial tension response to the sinusoidal interfacial area change. A typical plot of interfacial tension and area is shown in Figure S6 in the Supporting Information for 0.1% Star 52 in m-xylene against water at 0.2 Hz. The two curves show very little phase difference, indicating highly elastic behavior. It has been reported that nanoparticles with adsorbed polymer arms adsorbed at the oil/water interface can show nonsinusoidal behavior due to the polymer particles being ejected during the contraction of the interface, this was not the case here.^39^

Plots of the interfacial storage moduli and phase angles as a function of frequency are shown in Figure 9 for the Star 210, those for Star 52 and 55 are shown in the Supporting Information (Figure S8). Star 52 showed a small dependence of both storage modulus and phase angle on the frequency with the moduli in the range of moduli from ca. 14–21 mN m^–1^ and the phase angles below 10°. The small frequency dependence may have been a result of the limited equilibration time for this polymer. Star 55 showed an almost frequency-independent response with the moduli ranging from 10.5 to 14 mN m^–1^ over the range of concentrations and phase angles almost exclusively less than 5°. The storage modulus for Star 52 showed a small increase with concentration larger than the experimental error of ± 10%, whereas any variation seen with Star 55 with either frequency or concentration was within the experimental error. This data showed that these two polymers adsorbed at the interface to form highly elastic interfacial layers, which may be contrasted with the responses seen with Star 210. The storage moduli and phase angles for this polymer were dependent on both frequency and concentration, suggesting that the polymer was not as strongly bound at the interface as the other two. This may be attributed to the relative lengths of the PBA and PEO arms, as will be discussed later.

**Figure 9:**
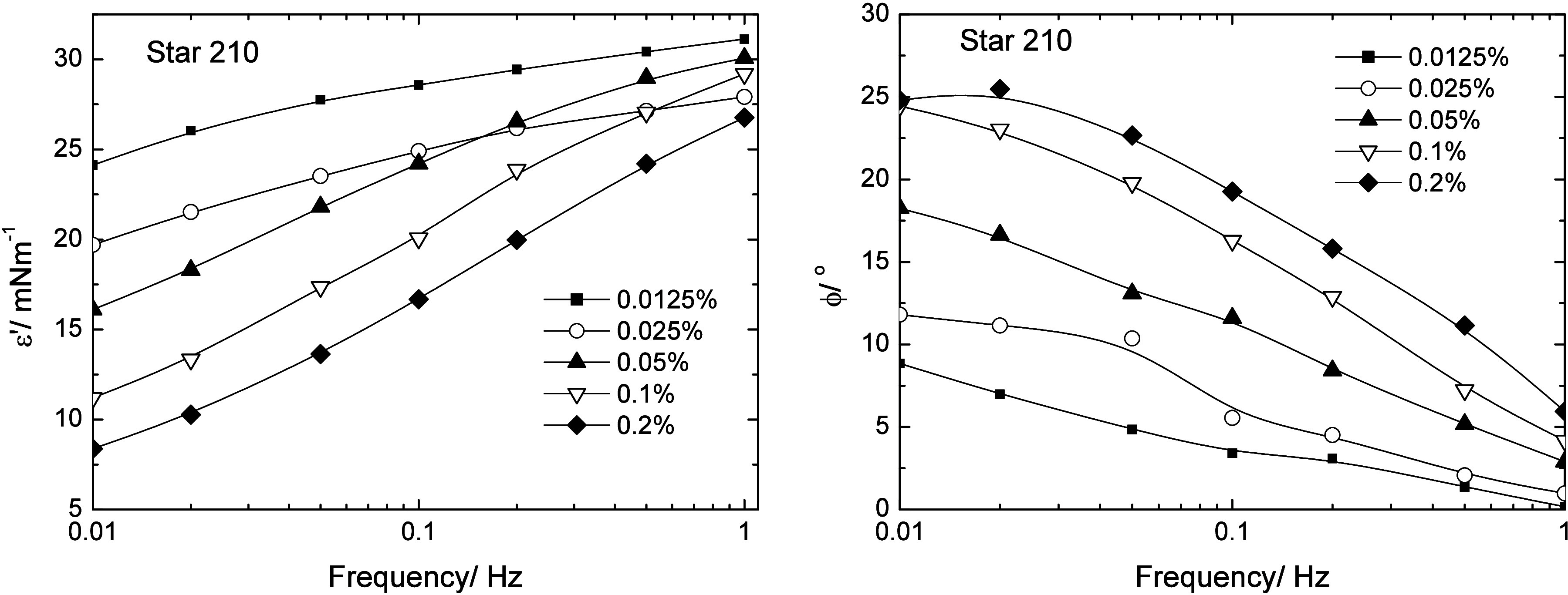
Interfacial moduli (left) and phase (right) for Star 210 adsorbed at the m-xylene/water interface. Error in moduli ± 10%.

The adsorbed layers of PLA–PEO polymer at the n-dodecane/water interface also gave highly elastic layers with phase angles generally less than 5°, and the storage moduli showed a small increase with frequency but was independent of concentration within experimental error (Figure S9, Supporting Information).

The rheological response of materials is dependent on the relaxation time, t_r_, of the system, which characterizes the time scale at which the material dissipates an applied stress or strain.^49^ In the case of interfacial rheology, the dissipation mechanism would be through changes in the adsorbed layer, either through adsorption/desorption of polymer particles or conformational changes that reduce the variation in interfacial tension during the oscillation process.^9,34,35,37,38^ In bulk rheological measurements, it has been found that plotting the moduli as a function of the reduced frequency, ωt_r_ (where ω is the angular frequency and t_r_ is the relaxation time), can collapse disparate data onto a single master curve.^49^ It has been seen that the frequency responses of adsorbed layers of homopolymer PEO star could be normalized in the same manner as for the interfacial tension.^9^ In that case, the frequency was normalized through division by c^2^ and the data for a range of polymer concentrations could be reasonably collapsed onto a master curve (allowing for experimental error). Those polymers showed the diffusion-controlled adsorption kinetics described above, and this was applied not only to the time scale of the surface or interfacial tension reduction but also to the relaxation time of the adsorbed layer. The interfacial tensions of the miktoarm polymers showed a good collapse of the data when the surface age was normalized by the concentration alone, and this normalization has been applied to the frequency-dependent moduli obtained here. Plots of the storage moduli as a function of f/c or f/c^2^ for Star 210 adsorbed at the m-xylene/water interface are shown in Figure 10. Both procedures reduced the spread of the data, with the f/c reduction showing the tighter collapse onto a single curve (allowing for experimental error in the moduli). The loss moduli (also shown in Figure 10) were also similarly reduced onto a master curve using both normalization procedures, although there was little difference in the tightness of the collapse.

**Figure 10:**
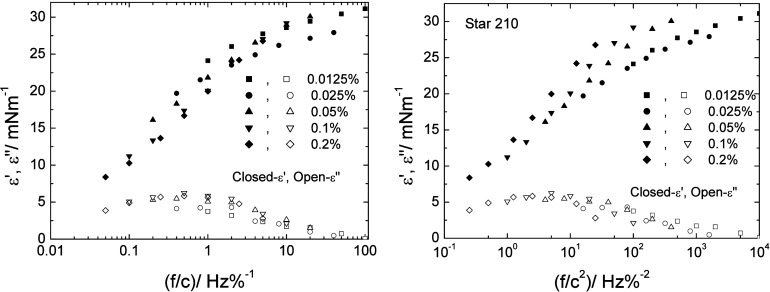
Plots of the interfacial storage and loss moduli for Star 210 adsorbed at the m-xylene/water interface as a function of reduced frequency, left- frequency normalized by the concentration, f/c, right- frequency normalized as for diffusion-controlled kinetics, f/c^2^. Error in moduli ± 10%.

The improved data reduction of the storage moduli seen with f/c again implies that any adsorption/desorption is not diffusion-controlled. The adsorbed layer of Star 210 showed a typical frequency response of adsorbed systems, with the storage moduli dominating at high frequencies where the time scale of the oscillation is too short for any significant relaxation process to occur. As the frequency is reduced, the oscillation time scale increases, allowing any relaxation processes to occur. At the lowest frequencies, the loss processes will dominate, and the response will become essentially viscous overall. This point had not been reached in the current data, and even at the lowest frequency at the highest concentration of Star 210 (0.2%), the storage modulus was still higher than the loss modulus. The relative lack of frequency dependence seen with Stars 52 and 55 and the PLA–PEO variant made any normalization unnecessary. The frequency dependence seen with Star 210 resulted from a short relaxation time attributed to a lower adsorption energy than the other three polymers. The rescaling of the data based on normalization by the concentration implies that the relaxation process is likely to be due to adsorption/desorption of the polymer particles during the oscillation, since these rates would depend on the concentration of the particles close to the interface.

A further test to determine whether the processes occurring during oscillation are diffusion-controlled or not is the variation of the static interfacial modulus ε_o_ given by eq 12:^50,51^

$$\varepsilon_{o}=\frac{\varepsilon^{\prime }{\left(f\right)}^{2}+\varepsilon^{\prime \prime }{\left(f\right)}^{2}}{\varepsilon^{\prime }\left(f\right)-\varepsilon^{\prime \prime }\left(f\right)}$$ 12

For a diffusion-controlled process, the calculated modulus is independent of frequency, and this was the case for a homopolymer PEO star with a PDVB core adsorbing at oil/water interfaces. The calculated static modulus, plotted in Figure 11 for Star 210, was frequency-dependent, confirming that the adsorption was not diffusion-controlled.

**Figure 11:**
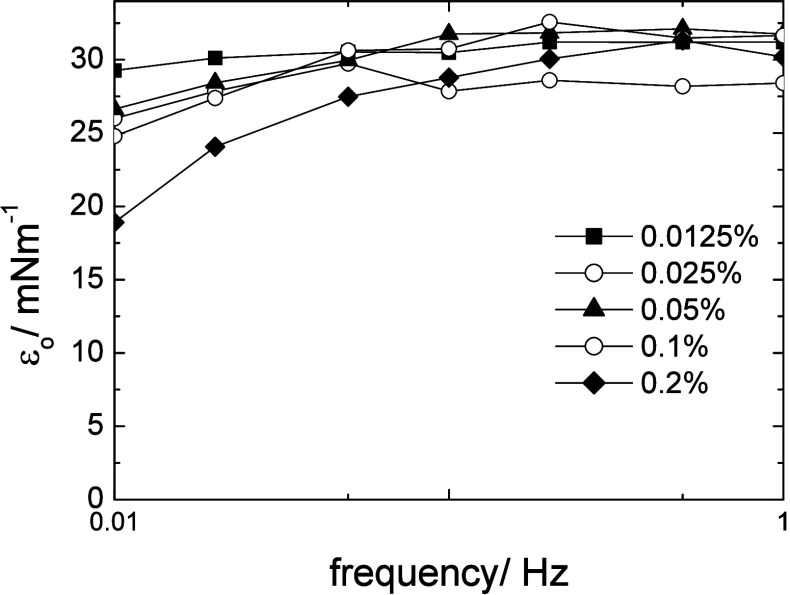
Variation in ε_o_ vs frequency at five concentrations for Star 210 adsorbed at the m-xylene/water interface. Error in moduli ± 10%.

The three PBA–PEO polymers also showed very different behavior regarding the size and stability of the m-xylene in water emulsions formed using them. The initial size distributions for the m-xylene in water emulsions obtained with these polymers are shown in Figure 12. To avoid significant Ostwald ripening arising from the relatively high m-xylene in water solubility, ca. 5% n-dodecane was added to each oil phase.^52^ The least efficient emulsifier was Star 52, which gave an emulsion with a peak diameter of 71 μm. Moreover, this emulsion was unstable against coalescence, with free oil being liberated within 2 days. Star 55 produced the overall smallest emulsion droplets, although the distribution was bimodal with peaks at 15.4 and 71 μm. Star 210 also gave a bimodal system, although the peaks overlapped to form a hump at higher diameter rather than a separate peak. The main peak occurred at 37 μm while the hump was centered around 100 μm leading to a larger overall volume mean diameter of 51 μm compared to 18 μm for Star 55.

**Figure 12:**
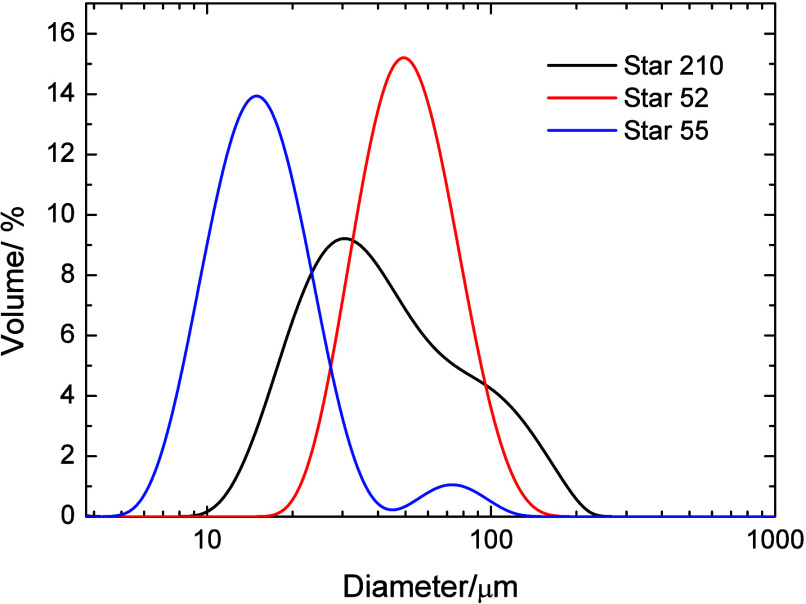
Initial droplet size distributions for m-xylene in water emulsions stabilized by Stars 52, 55 and 210.

The emulsions stabilized by Stars 210 and 55 showed growth over a period of 22 days at 20 °C, although no free oil was apparent. The size distributions obtained are shown in Figure 13, and both systems show a general move to larger diameters. In the case of Star 55, the lower diameter peak showed a small initial shift to a higher diameter (17.5 μm), and droplets within these peaks were undergoing coalescence, leading to an increasingly significant peak around 70–80 μm. The position of the upper peak showed a less clear trend with time. The sampling of very large emulsion droplets can be variable due to large creaming rates, leading to skewed results, and care was taken here to ensure the emulsions were as well dispersed as possible (the sample was inverted several times immediately prior to sampling). Despite these precautions, some sampling error was seen with the 8 and 22-day size distributions with Star 210, with the 8-day data showing the larger size. However, this does not affect the interpretation of the data, that being that Star 210 gave relatively unstable emulsions while Star 55 gave the most stable of the three systems, while still showing droplet coalescence over time.

**Figure 13:**
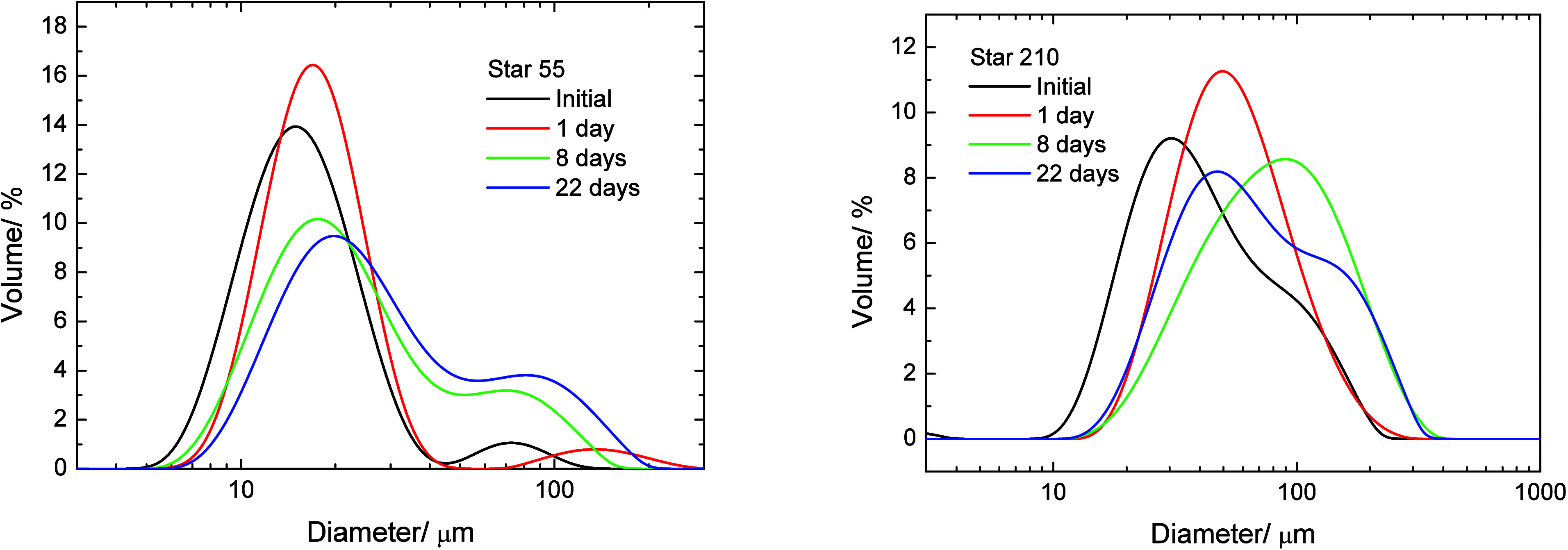
Evolution of the droplet size distribution with time for m-xylene in water emulsions stabilized by 0.1% Star 55 (left) and Star 210 (right).

The relatively small frequency dependence in the interfacial moduli and the interfacial layer wrinkling seen of droplet deflation seen with Stars 52 and 55 and with the PEO–PLA variant suggests that the particles were more strongly adsorbed than those of Star 210, which showed significant frequency and concentration dependence. For Stars 52, 55, and 210, the same ratio of EO and BA monomer (equal mass) was used for each of the variants. As such, the overall relative hydrophobicity of the three polymers was the same; despite this, two of the polymers showed much stronger adsorption than the third. The differences arise from the spatial distribution of EO and BA units in the corona of grafted arms surrounding the PDVB core.

Although the star polymers are all based on PDVB cores, the nature of the core will be different due to the PBA (or PLA) grafted arms forming an extended hydrophobic core, Figure 14. Star 52 essentially has an extended hydrophobic core, with the 2k PBA arms being more prevalent than the 5kPEO arms. Since there are 2.5 times more PBA arms than PEO arms, the layer of grafted arms close to the surface of the core is the most hydrophobic. The paucity of hydrophilic arms means that this extended core would have the highest water contact angle and reside more in the m-xylene phase. This accounts for the low stability of the O/W emulsions formed with this polymer since hydrophobic particles tend to stabilize W/O emulsions more effectively than O/W.

**Figure 14:**
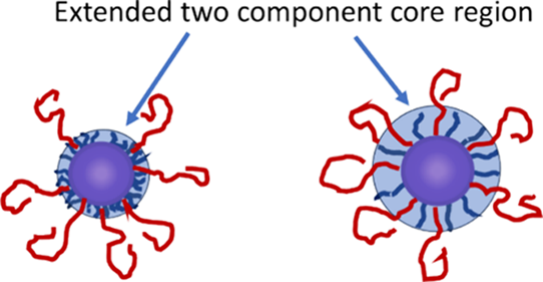
Extended hydrophobic cores of Star 52 (right) and Star 55 (left).

Star 55 has a more extended hydrophobic core owing to the PBA arms being somewhat longer than Star 52. Since there are now an equal number of hydrophilic and hydrophobic arms (each arm having the same total molecular weight), the extended core is less hydrophobic and will have a lower water contact angle than that of Star 52. The larger extended core radius and the lower water contact angle will result in higher adsorption energy, and the particle will penetrate further into the water phase than Star 52. This results in the observed enhanced against coalescence in the O/W emulsions.

Applying this approach to Star 210, the extended core may be viewed as being the PDVB core and the complete corona of grafted arms, since the PEO and PBA have similar contour lengths. In this case, the particle will act more like a simple, relatively hydrophilic particle since there are five times more PEO arms than PBA. As such, the particle will favor the aqueous phase over the m-xylene to a greater extent than Star 52 or 55, leading to lower adsorption energy that is exacerbated by the smaller overall combined radius and low interfacial tension for this polymer. Thus, the properties of Star 210 would be expected to be closer to those of homopolymer PEO star, and this is the case in terms of the interfacial moduli, where a significant frequency dependence has been seen here and for 2k PEO star polymers with a PDVB core. This interpretation of an extended core is similar to that suggested by Reed et al. for grafted poly (glyceryl methacrylate) arms on polystyrene latex particles.^55^ Their data suggested that sufficiently long PGMA arms formed a compact hydrated layer in water, leading to a low water contact angle despite relatively low grafting densities. In the present case, it would be the PBA arms that would be forming a solvated layer that increases the effective diameter of the core. It also should be noted that the compressibility of the PDVB core is unknown and may be softened by the m-xylene. For the present analysis it is assumed to be a well-defined spherical core and it is envisaged that any softness would not significantly affect the conclusions, especially as any softening would be comparable for the three PBA–PEO systems. The predicted positions of the adsorbed polymers at the m-xylene/water interface are shown in Figure 15.

**Figure 15:**
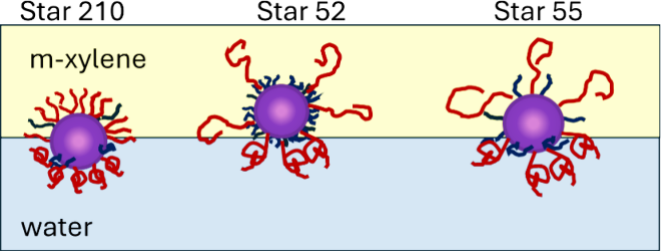
Predicted positions of Star 210, 52, and 55 adsorbed at the m-xylene/water interface. The penetration of the PEO chains into both phases will tether the particles at the interface.

It should be noted that the conformations of the PBA and PEO arms were not included in the discussion of the extended cores, although the different conformations would change the thickness of the extended core. Although this would be the case for the PBA and PLA since these would be expanded in the oil phase but collapsed in the aqueous phase, this would not qualitatively change the arguments. The star polymers are interesting in that they have the potential act differently to a simple solid particle of a similar size and wettability of the extended star core in that they may show a composite adsorption mechanism resulting from differing hydrophilic and hydrophobic arm lengths. In addition to the adsorption energy arising from the extended core another mechanism may be acting to hold polymer at the interface. The PEO chains in both Stars 52 and 55 are significantly longer (in terms of contour length of the backbone) than the hydrophobic PBA arms.^44^ This allows the PEO arms to penetrate both the m-xylene and aqueous phases while the more hydrophobic PBA arms remain close to the core surface in the aqueous phase. This, in combination with the larger effective hydrophobic core and associated increased adsorption energy, would effectively tether the particles at the interface and would not readily desorb when the interface is contracted under oscillation and the slow adsorption kinetics seen in the interfacial tension measurements would prevent significant adsorption during the interfacial expansion. In contrast, the arms in Star 210 are all of similar length and so act as more of a particle with a composite surface of hydrophilic and hydrophobic parts with no significant penetration of individual PEO arms into either of the two phases.

The tethering effect is slightly modified for the PLA–PEO analogue, since n-dodecane is a worse solvent for PEO than m-xylene. In this case, the lauryl chains will be better solvated than the PEO in the oil phase but fully collapsed on the aqueous phase side of the interface. In contrast, the PEO will be extended in the aqueous phase but less so in the n-dodecane. The radius of gyration of PEO is known to be less in the very similar solvent *n*-heptane as discussed by Hezaveh et al.^45^ In some ways, this polymer would then act more as an amphiphilic particle with a high adsorption energy.

Transfer to or from the interface and conformational changes in the adsorbed polymer during the oscillation may contribute to the viscous or loss component of the dilatational modulus. In the absence of these, the response would be dominated by the elastic response as the interfacial tension increases and decreases as the adsorbed interfacial layer is expanded and contracted during oscillation. This system would not show frequency dependence in the moduli and a low phase angle. Stars 52 and 55 as well as the PLA–PEO variant fall into this category as a result of the effective hydrophobic core and the tethering effective of the PEO chains. Facile transfer to and from the interface allows particles to adsorb and desorb during the oscillation, reducing the magnitude of the change in interfacial tension. This also leads to a frequency dependence for both the viscous and elastic moduli, and the phase angle depends on the applied frequency and the characteristic time scale of the adsorption/desorption process. Star 210 falls into this category, sitting at the interface as a relatively more hydrophilic particle with shorter PEO chains, giving a reduced tethering effect, leading to faster adsorption kinetics and some degree of desorption/adsorption during the oscillation measurement, leading to a frequency-dependent response. The adsorbed layers of this polymer also showed much higher phase angles than the other polymers.

It is known that conformational changes in adsorbed polymers will also determine the dilatational response. For example, the promotion of distal segments far from the interface into the proximal region will lead to an enhanced viscous response.^35^ This does not seem to be a factor in the present case in that Star 55, with longer PEO chains, would be expected to show this effect to a greater degree than Star 210 and to have a greater frequency dependence and phase angle. This is contrary to that seen with these star polymers and as such the dilatational responses are more strongly determined by adsorption/desorption.

The interfacial moduli are also governed by the change in interfacial tension with the change in the area occupied by particles or polymer molecules (eq 1). For a given change in adsorbed area per particle, closely packed adsorbed particles showing hard lateral interactions that are strongly dependent on particle separation, the change in interfacial tension will be higher than those with softer, more long-range interactions, which vary more slowly with separation. This has been seen with pH-dependent systems in which star-type polymers show greater moduli in the uncharged state (harder interaction) than in the charged state (softer interaction).^53^ Stars 52 and 55 and the PLA–PEO polymer, by virtue of their longer PEO arms will pack less densely and show a relatively soft interaction that leads to the lower moduli seen here compared to that for Star 210. With its more compact structure of short PEO and PBA arms, Star 210 will show a harder interaction which, in conjunction with the closer packing, will give higher moduli. The more facile adsorption/desorption seen with this polymer will offset this to some extent.

The equilibrium interfacial tensions seen with the three polymers depended on the molecular masses of the individual arms. The lowest interfacial tension was obtained with Star 210 and the highest with Star 55. The interfacial tension will be a function of the interfacial excesses of the polymers, their lateral interactions, and their interfacial free energies. The interfacial excesses were not known, but it is reasonable to assume that Star 210 would show the greatest density of packing of the core particles at the interface due to having the shortest arms, leading to a low interfacial tension. Both Star 52 and 55 would adsorb fewer particles per unit area as a result of the steric interactions between their longer PEO arms and so show higher interfacial tensions. The adsorption of a homopolymer PDVB–PEO star with 5k arms gave an interfacial tension of 17.5 mN m^–1^ at the m-xylene/water interface and was comparable to that obtained here with Star 55 (16 mN m^–1^) while Star 52 showed a slightly lower value of 10–13 mN m^–1^. In contrast, Star 210 showed a much lower interfacial tension (4 mN m^–1^) than the homopolymer 2k PEO star (15 mN m^–1^) at the m-xylene/water interface and the reason is not clear since they might be expected to show similar packing. The interfacial tension is a complex result of many factors, and without further study, it is not possible to explain the difference seen between Star 210 and the 2k PEO homopolymer variant, especially in view of the likely difference in the number of arms per particle. For instance, the effect of the combination of the PEO and PBA on the interfacial free energy is not known. It is also likely that the extent of the penetration of the hydrophobic core will also be a factor, as that will determine the area of unfavorable oil/water interface lost through the adsorption of the particle. It appears that from the comparison of these homopolymer and miktoarm stars at the m-xylene/water interface that a combination of hydrophilic and hydrophobic arms at the interface is potentially more effective at reducing the free energy than hydrophilic arms alone. However, this is not the case with the PLA–PEO variant at the n-dodecane/water interface. This showed little concentration dependence, ranging from 24.4 to 26.6 mN m^–1^ and was comparable to that seen with homopolymer 5k PEO star with DVB cores at the same interface (26 mN m^–1^). Further work is required to determine the factors underlying the interfacial tensions in these systems.

The longer PEO arms of Stars 52 and 55 will result in a lower density of adsorbed particles owing to the longer range, softer steric interactions. Compared to Star 210, these would result in smaller lateral interactions between the adsorbed polymers. The weaker interactions and lower adsorbed amount would lead to a smaller reduction in interfacial energy in terms of the bare oil/water interface destroyed compared to Star 210. The difference between Star 52 and 55 may be viewed in similar terms to an alcohol ethoxylates adsorbed at the oil/water interface and their hydrophilic lipophilic balance (HLB), which is a measure of the relative hydrophobicity and hydrophilicity of a surfactant. The higher the HLB, the more hydrophilic the ethoxylated surfactant. Longer chain PEO higher HLB surfactants tend to give higher interfacial tensions than those with shorter chains (lower HLB) for the same hydrophobe. The analogy is not exact but the greater fraction of PEO arms in Star 55 would give a greater effective HLB for the particle and so would give a higher interfacial tension. Further work is required to determine the factors underlying the interfacial tensions in these systems.

## Conclusions

The results obtained in this work highlight the importance of the spatial distribution of hydrophobic and hydrophilic segments within a star polymer in which the arms are a blend of solely hydrophilic and solely hydrophobic chains. Three of the polymers reported here show the same ratio of the same hydrophobic and hydrophilic segments but show differences in the relative molecular masses of arms. Despite having the same overall hydrophobicity, the three polymers show distinctly different interfacial properties. Further systematic studies of the effect of graft density and arm molecular weights are required to understand better the nature of the adsorption of star polymers at oil/water interfaces.

## Supplementary Material



## Notes

GPC traces and molecular weights for the four mikto arm polymers; ^1^H NMR trace for Star 210; discussion of droplet deformation and accuracy of interfacial tension measurement; time dependence of the interfacial tensions of Star 52 and of the PLA–PEO analog; normalization of interfacial tension vs time for Star 52 and the PLA–PEO analog, typical variation of interfacial tension with droplet area in dilatation measurement; frequency dependence of the interfacial moduli and phase angle for Stars 52 and 55 and the PLA–PEO analogs (PDF)
