# Supplementary material for: Interfacial Properties of Miktoarm Star Polymers with a Poly(divinylbenzene) Core
Source: Langmuir. 2025 Jul 14;41(29):19023–36. doi: 10.1021/acs.langmuir.5c00288 (PMC12312146; doi:10.1021/acs.langmuir.5c00288)
Supplement: Supplementary file 1 [file la5c00288_si_001.pdf]

Supporting Information:

Interfacial Properties of Miktoarm Star Polymers with a Poly(Divinylbenzene) Core

Ting-Chih Lin[a], Mateusz Olszewski[a], Jiajun Yan[a,b], Xiaolei Hu[a], Krzysztof Matyjaszewski [a,c]*, Philip Taylor[d]*

a. Department of Chemistry, Carnegie Mellon University, Pittsburgh, PA 15213, USA.
b. School of Physical Science and Technology, ShanghaiTech University, Pudong, Shanghai, China 201210
c. Department of Molecular Physics, Faculty of Chemistry, Lodz University of Technology, Żeromskiego 116, 90-924 Łódź, Poland
d. Syngenta, Jealott's Hill International Research Centre, Bracknell, Berkshire, RG42 6EY

*Corresponding authors

This document details additional information concerning the paper "Interfacial Properties of Miktoarm Star Polymers with a Poly(Divinylbenzene) Core" and includes molecular weight data, analysis of the accuracy of the interfacial tension and moduli measurements and interfacial tension and rheology results.

1. Star polymer characterization
   Molecular weight distributions for the four star polymers- determined by GPC using THF eluent at a flow rate of 1 mL/min at 35˚C and linear PMMA as the calibration standards.

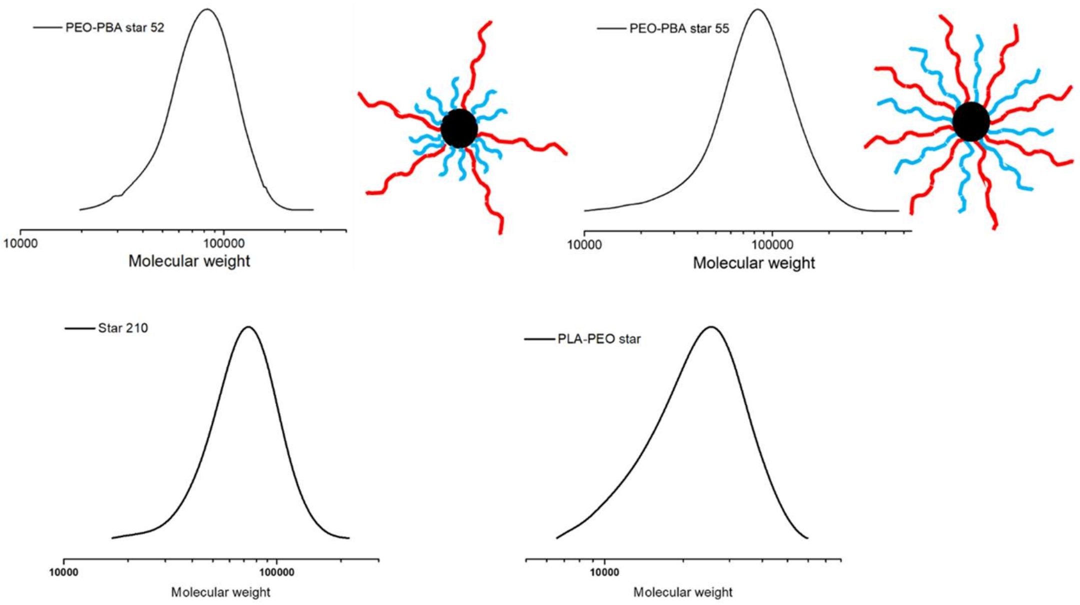

Fig S1: GPC molecular weight distributions for Stars 210, 52 and 55 and the PLA-PEO analog

Table S1. GPC data in THF eluent for star samples and synthesized macroinitiator arms.

**Sample**	**Arms**	**M_w(g/mol)**	**M_w/M_n**
Star55	PEO 5k/PBA 5k	8.9×10^4	1.27
Star52	PEO 5k/PBA 2k	8.3×10^4	1.15
Star 210	PEO 2k/ PBA 10k	7.4×10^4	1.14
PLA-PEO	PEO 5k/ PLA 6k	2.5×10^4	1.17

It should be noted that molecular weights listed in Table 1 and Table S1 represent apparent values based on poly(methyl methacrylate) standards for consistency with our previous publication.[1] However, the absolute values of molecular weights determined using a multiangle light scattering detector for very similar stars are typically ca. 4 times larger, as reported in literature.[2-4] This suggests that the actual molar mass of the core should also be ca. 4 times larger and the radius of the core 1.58 times ($4^{1/3}$) larger and the surface area of the core 2.5 times ($4^{2/3}$) larger than values reported in Table 1. This would also correspond to 4 times larger number of arms and 1.58 times smaller areas per arm than based on apparent molar masses in Table 1.

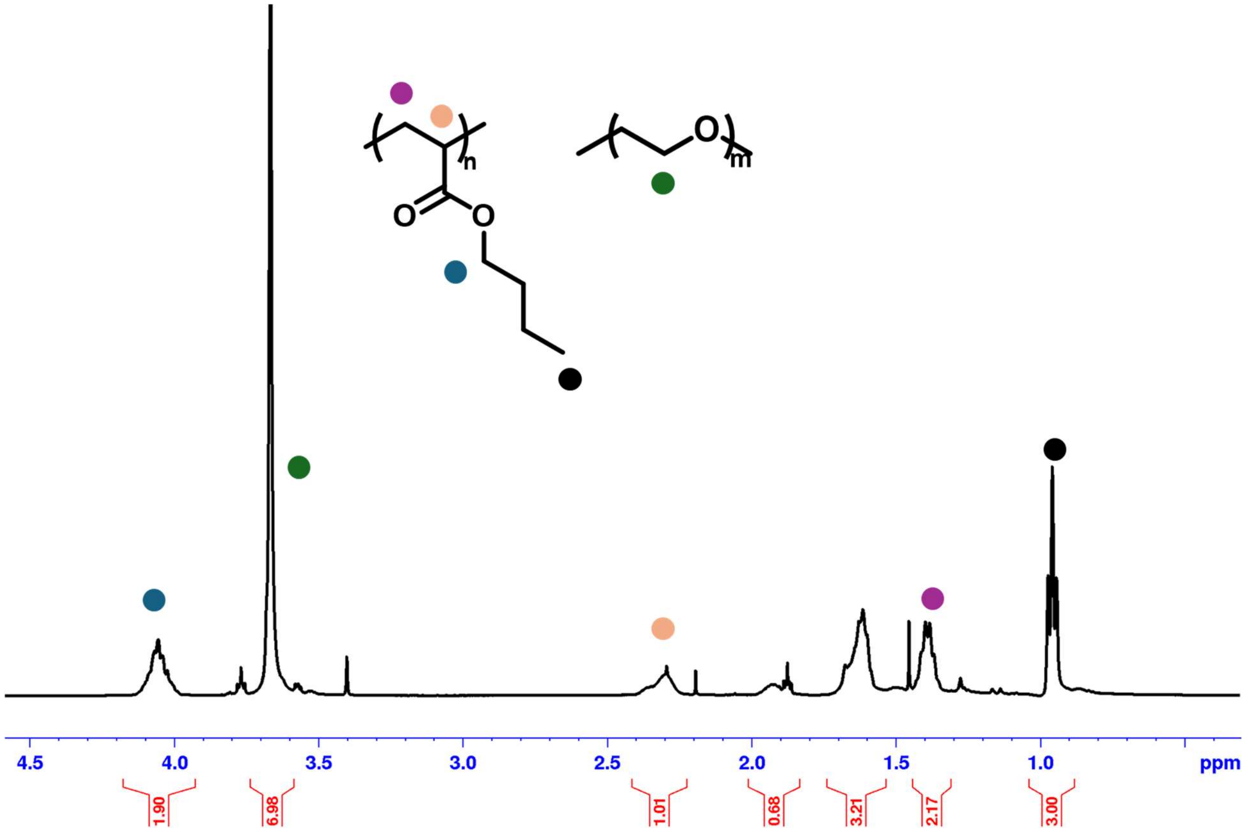

Fig. S2. ^{1}H NMR spectrum of Star 210 recorded in $CDCl_3$. It was used to confirm the agreement of the chemical composition of the arms in the star with the initial feed of macroinitiators.

2. Droplet deformation and accuracy of the extracted interfacial tensions

The accuracy of the extraction of the interfacial tension from the droplet profile depends on the extent of the deformation of the droplet. This deformation depends on the droplet volume, interfacial tension and density difference between the two phases. If the droplet deformation is small and the droplet is close to spherical, then the extracted interfacial tension is likely to be inaccurate, as has been found by Berry.[5] The deformation may be considered in terms of the bond number, which is a dimensionless number that quantifies the ratio of gravitational to interfacial tension forces. Berry et al. extended this approach and defined a new number called the Worthington number for pendant drops, given by the ratio of the droplet volume compared to the maximum volume at which the droplet detaches under gravity.[5] The Worthington number, W, is given by equation S1:

$$Wo = \frac{\Delta\rho g V_d}{\pi\gamma D_n} \qquad \text{S1}$$

Where $\Delta\rho$ and γ are the density difference and interfacial tension between the two phases, V_d is the droplet volume and D_n is the needle outer diameter. Berry et al. found that to give an accuracy of better than 0.2 mNm^{-1} a Worthington number in excess of ca. 0.58 was required, in this region, the standard error across a data set was better than 1%. [5]

The droplet volumes used in the current study were of the order of 3.3-7.2 mm^3 and for Stars 210, 52 and 55 the needle outer diameter was 0.5mm. The small density difference between water and m-xylene resulted in a smaller deformation than if the droplet was in air, but this was offset in the case of Star 210 by the low interfacial tension of ca. 4 mNm^{-1} which resulted in Worthington numbers of 0.75-0.83 and so was well in the most accurate region. Typical Worthington numbers for Stars 52 and 55 were mostly below the optimum value, although Star 52 was close to 0.58 at the two highest concentrations. However, it is thought that the droplets were sufficiently deformed to make reasonable interfacial tension determinations. It should be noted that the paper by Berry et al. was not known to us at the time of this work, had it been so then the droplet size would have been increased for Stars 52 and 55. However, the slow reduction in interfacial tension caused uncertainty in judging the final deformation. Moreover, since the volume was increased during the oscillation, it was necessary to ensure that the droplet did not detach during these measurements. This was a problem with Star 210 due to its low interfacial tension.

The Worthington number for the PLA-PEO analog at the n-dodecane/ water interface was in the range 0.48-0.5 for all of the concentrations used, despite the measurement being made with a 1.65mm outer diameter needle. This difference was offset to a great extent by the larger density difference of 0.25 g cm^{-3} compared to the 0.14 g cm^{-3} for water/ m-xylene.

Table S2: Calculated Worthington numbers for the PBA-PEO based miktoarm star polymers

	Star 210			Star 52			Star 55		
Conc/%	*g*	V_d/mm^3	Wo	g	V_d/mm^3	Wo	g	V_d/mm^3	Wo
0.01				13.1	6.3	0.42	16.4	6.3	0.34

0.0125	*3.8*	3.3	0.75						
0.025	4.05	3.6	0.96	12.3	5.9	0.42	15.9	7.2	0.39
0.05	*4.4*	3.7	0.74	12.3	5.5	0.39	16.1	7.0	0.38
0.1	4.35	4.0	0.79	10.0	6.4	0.56	16.1	6.9	0.37
0.2	*4.1*	3.9	0.83	9.7	6.5	0.59	16.3	6.8	0.36

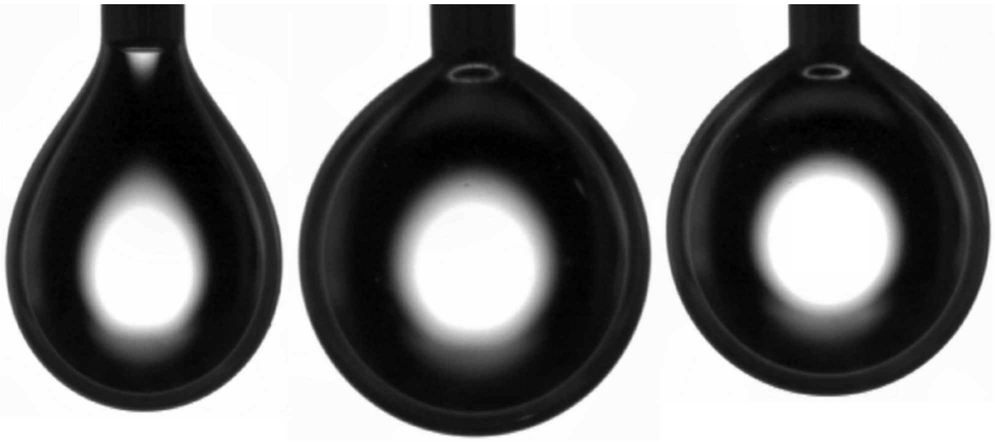

Figure S3: Representative droplet profiles for 0.025% Star 210 (left), Star 52 (centre) and Star 55 (right).

However, despite the potential uncertainties in the interfacial tension measurements for the 3 polymers, no obvious issues arose from the repeatability of the measurements on a given sample.

Berry et al. considered the effect of the Worthington number on the measured surface tension of water for a number of needle diameters and drop volumes.[5] Their data for a needle with an outer diameter of 0.51 mm shows that at Wo=0.3 the measured surface tension is ca. 1-1.5 mNm^{-1} below the true value of 72.4 mNm^{-1}, whilst at Wo=0.4 the difference is less than 1 mNm^{-1}. Scaling these differences down to the 16 mNm^{-1} found for Star 55 gives a likely error of 0.3 mNm^{-1} at Wo=0.34. This was of sufficient accuracy for the arguments presented in the accompanying paper.

It was noted that a reduced interfacial tension of 14.4 mNm^{-1} was obtained for a 3.3 mm^3 droplet with a Worthington number of 0.2 for 0.1% Star 55. This was in agreement with Berry et al., who reported the extracted interfacial tension began to decrease significantly below Wo=0.3. Similarly, for 0.2% Star 55, a droplet of 5 mm^3 and Wo of 0.27 gave a reduced interfacial tension of 15.4 mNm-1 compared to 16.3 mNm-1 with a 7 mm^3 droplets of Wo=0.6. The consistency of the interfacial tensions obtained for all concentrations of Star 55 suggest that the droplets with Wo in the range 0.34-0.39 were sufficiently distorted to give sufficiently reproducible data. Overall, it was concluded that measurements made on droplets with Wo of ca. 0.34 and above gave consistent reproducible values within an error of 0.5 mNm^{-1} over the whole interfacial tension range tested.

3. Time dependence of the interfacial tension of Star 52 and the PLA-PEO analog at the m-xylene/ water and n-dodecane/water interfaces, respectively.

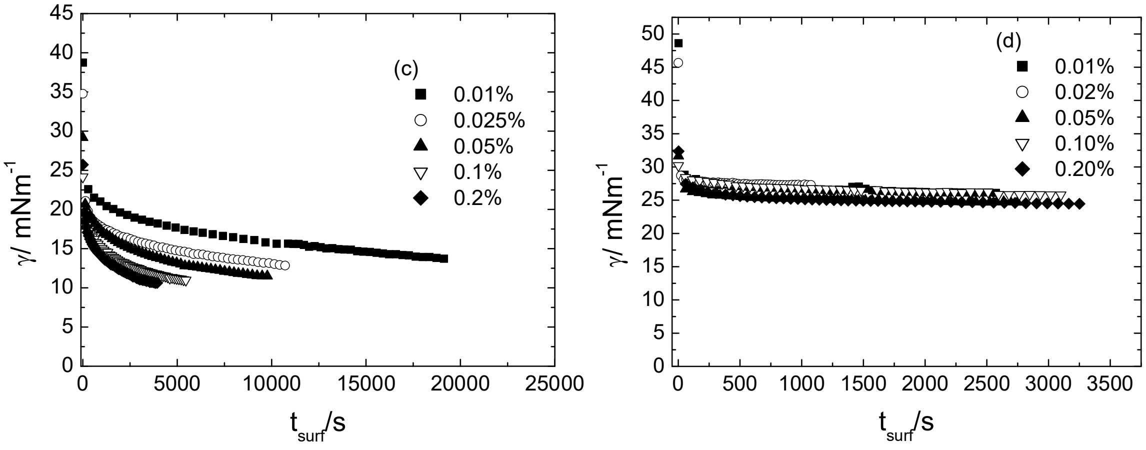

Figure S4. Time dependence of the interfacial tension for miktoarm star polymers at the oil/ water interface, left Star 52 vs m-xylene (c), right star PLA-PEO vs n-dodecane (d). Error in interfacial tension ±0.5 mN m^{-1}.

Star PLA-PEO showed little dependence of interfacial tension on concentration at the n-dodecane/ water interface (Figure S5)

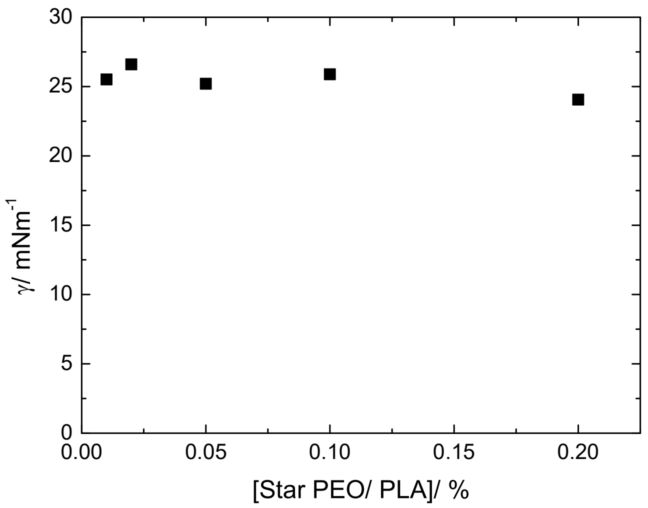

Figure S5: Plot of equilibrium interfacial tension versus concentration for star PLA-PEO at the n-dodecane/ water interface

4. Normalization of interfacial tension vs time data

Figure S6 shows the interfacial tension vs time data for different concentrations of Star 52 and the PLA-PEO analog normalized by multiplication of the time by the concentration.

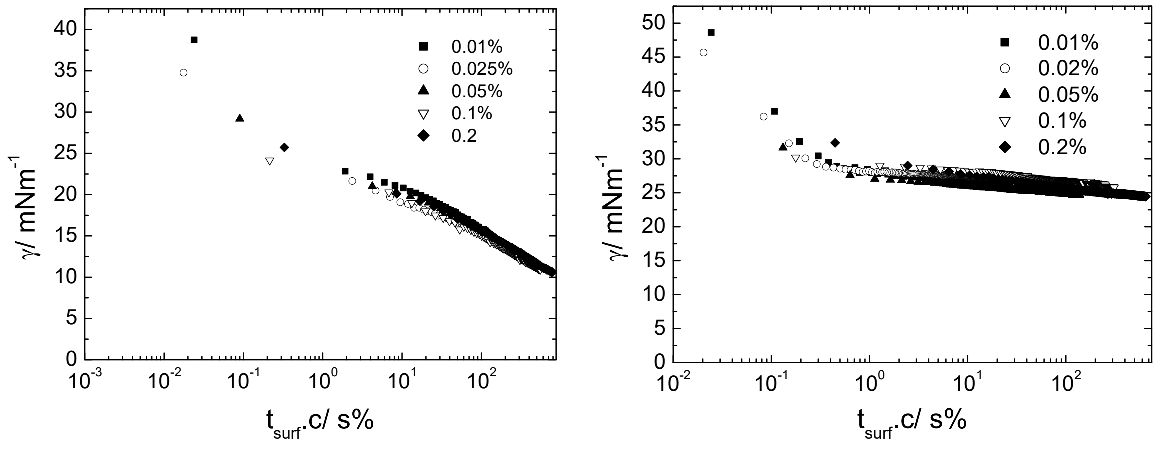

Figure S6. Interfacial tension time versus time normalized by the concentration for Star 52 at the m-xylene/ water interface (left), and the PLA-PEO variant at the n-dodecane/ water interface (right). Error in interfacial tension ±0.5 mN m^{-1}.

5. Droplet area and interfacial tension versus time

Figure S7 shows the variation in interfacial tension and droplet surface area for a droplet of water in a 0.1% solution of Star 52 in m-xylene. The data demonstrates the interface was highly elastic in nature with a small phase difference between the two curves. The almost pure sinusoidal interfacial tension response suggests any error due to a low Worthington number was negligible.

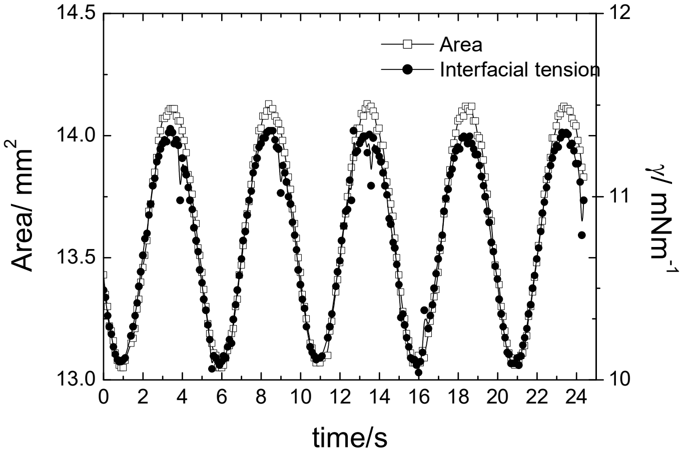

Figure S7. Interfacial tension and interfacial area as a function of time during an oscillation measurement on 0.1% Star 52 at the m-xylene/ water interface.

6. Frequency response of the interfacial properties

Figure S8 shows the variation of the interfacial moduli (ε') and phase angle (f) with frequency for Stars 52 and 55. The moduli showed relatively little variation with frequency and the low phase angles indicated highly elastic interfacial adsorbed layers

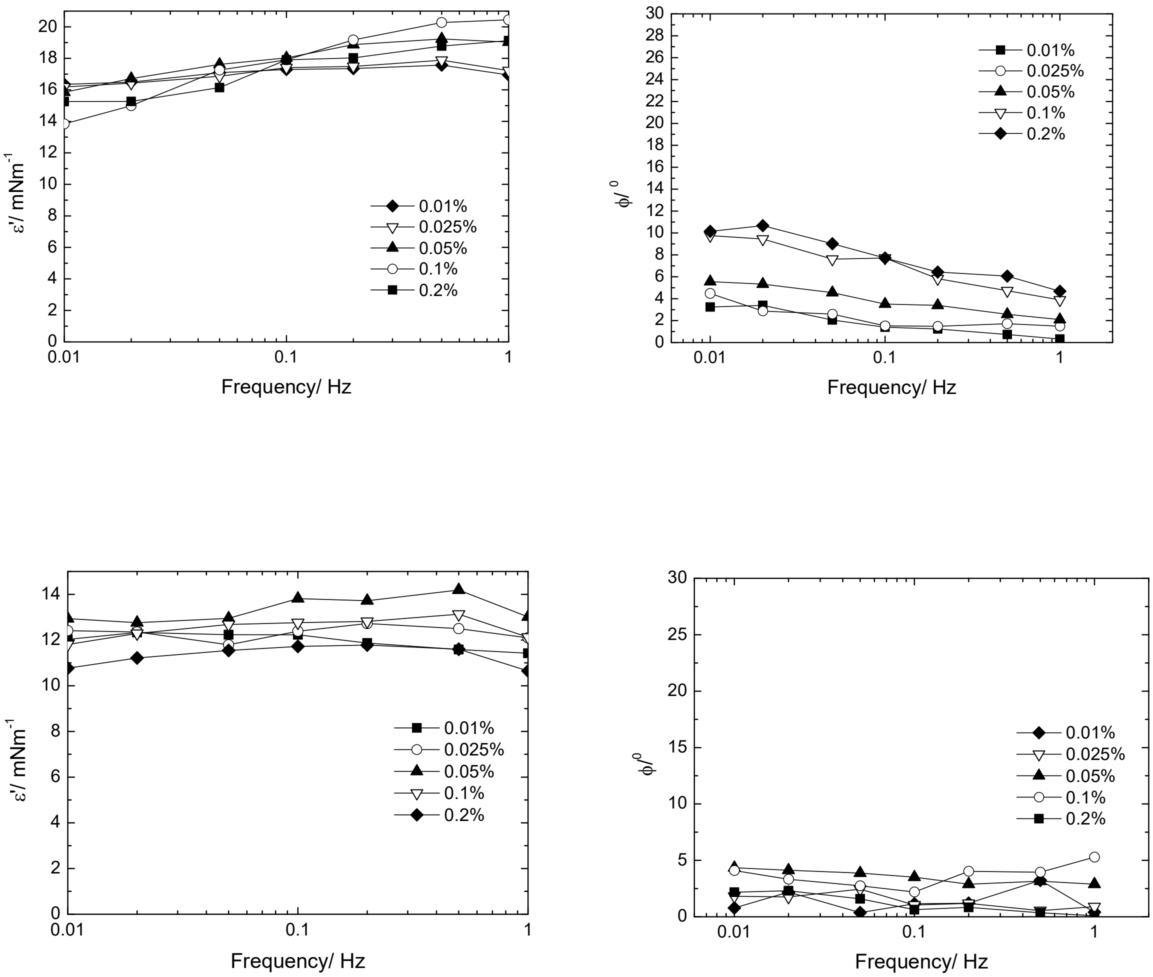

Figure S8. Interfacial storage moduli (left) and phase angle (right) for (top to bottom) Stars 52 and 55 adsorbed at the m-xylene/ water interface. Error in moduli ±10%.

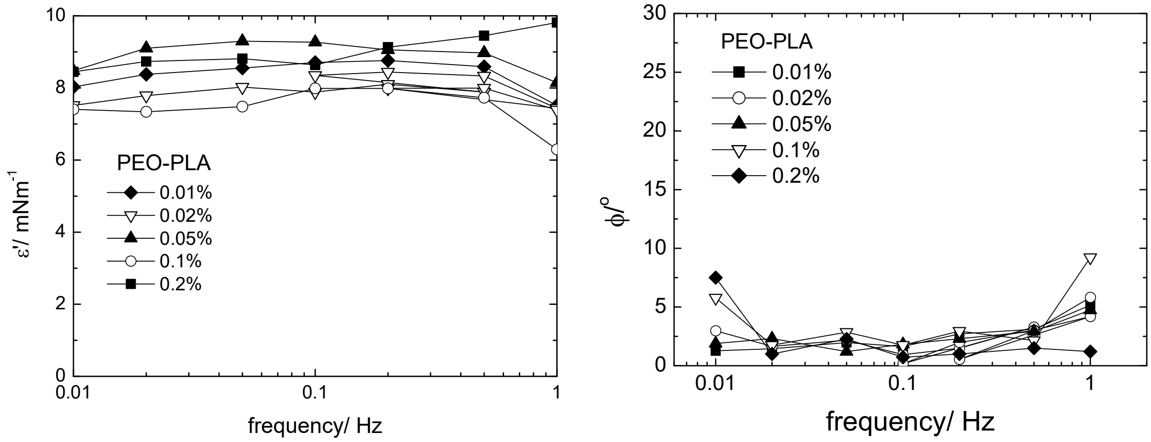

Figure S9. Interfacial moduli (left) and phase angle (right) for the PLA-PEO star polymer adsorbed at the n-dodecane/ water interface. Error in moduli ±10%.

References

1. Olszewski, M.; Hu, X.; Lin, T.-C.; Matyjaszewski, K.; Lebedeva, N.; Taylor, P., Oscillatory and Relaxation Study of the Interfacial Rheology of Star Polymers with Low-Grafting-Density PEO Arms and Hydrophobic Poly(divinylbenzene) Cores. *Langmuir* **2023,** *39*, 7741-7758, DOI: 10.1021/acs.langmuir.3c00557
2. Li, W.; Yu, Y.; Lamson, M.; Silverstein, M. S.; Tilton, R. D.; Matyjaszewski, K., PEO-Based Star Copolymers as Stabilizers for Water-in-Oil or Oil-in-Water Emulsions. *Macromolecules* **2012,** *45*, 9419-9426, DOI: 10.1021/ma3016773
3. Gao, H.; Matyjaszewski, K., Arm-First Method As a Simple and General Method for Synthesis of Miktoarm Star Copolymers. *J. Am. Chem. Soc.* **2007,** *129*, 11828-11834, DOI: 10.1021/ja073690g
4. Gao, H.; Matyjaszewski, K., Synthesis of Low-Polydispersity Miktoarm Star Copolymers via a Simple "Arm-First" Method: Macromonomers as Arm Precursors. *Macromolecules* **2008,** *41*, 4250-4257, DOI: 10.1021/ma800618d

5. Berry, J. D., Neeson, M. J., Dagastineb, R., R., Chan, D. Y. C., Tabor, R. F., Measurement of surface and interfacial tension using pendant drop tensiometry, *J. Colloid Interface Sci*., **2015**, 454, 226-237, https://doi.org/10.1016/j.jcis.2015.05.012
